# When Do Skeletal Class III Patients Wear Their Reverse Pull Headgears?

**DOI:** 10.1155/2017/3546262

**Published:** 2017-03-09

**Authors:** Nurhat Ozkalayci, Orhan Cicek

**Affiliations:** Department of Orthodontics, Faculty of Dentistry, Bulent Ecevit University, 67100 Zonguldak, Turkey

## Abstract

*Objective*. The aim of this study is to evaluate the factors that affect wearing time and patient behavior during reverse pull headgear therapy with a newly designed reverse pull headgear.* Methods*. In clinical practice, new reverse pull headgears were applied to fifteen patients. The patients were monitored during reverse pull headgear therapy and the data were evaluated. Statistical analysis was made.* Results*. During the study, patients were monitored successfully and the evaluations showed that patients wear the new reverse pull headgears mostly at night. There are differences between days of week and hours of day. Weekends are more popular than weekdays for wearing reverse pull headgear.* Conclusions*. This new type of reverse pull headgears can be used successfully in clinical practice and can help the clinician. Study showed that the most important factor that affects the cooperation of reverse pull headgear patient is aesthetic appearance.

## 1. Introduction

Maxillary deficiency, mandibular prognathism, or both can lead to skeletal class III malocclusion. Different orthodontic approaches used to treat maxillary deficiency include early orthopedic correction, fixed treatment, or a combination of fixed mechanics and surgery. Early orthopedic correction can be done at the proper ages using extraoral appliances like reverse pull headgear [1]. Reverse pull headgear therapy is the gold standard for correcting maxillary deficiency and achieving maxillary protraction, but its effectiveness depends on the amount of time and regularity that patients wear reverse pull headgear [2, 3]. Objective and strict observation of reverse pull headgear therapy is needed and may help improve the level of treatment success.

Some studies have been done on patient compliance during different orthodontic protocols such as intraoral appliance therapy [4, 5]. Only a few devices are available to quantify extraoral appliance wearing time and usage regularity [6, 7].

Aim of this study is to present the objective evaluation of the wearing time of a reverse pull headgear during therapy.

## 2. Materials and Methods

A total of 15 patients (8 Males, 7 Females; mean age 11.9 ± 0.9 years; age range 11–13 years) with maxillary deficiency and skeletal class III were included in the study. Pretreatment orthodontic records were taken from all patients and evaluated. An expert orthodontist evaluated hand wrist radiographs and clinical signs of patients.

Inclusion criteria of the study were active growth period, true class III skeletal relationships due to maxillary deficiencies, a negative ANB angle due to lowered SNA angle, posteriorly positioned A point due to maxillary deficiency according to McNamara analysis, and concave profile.

All of the patients were appropriate for early orthopedic correction according to their age and hand wrist radiographies and evaluations of an expert orthodontist.

Ethical approval was obtained from the Ethical Commission of the University. All patients were informed about the treatment method and reverse pull headgear therapy. Patients also were informed about the iButtons placed on the forehead part of the newly designed reverse pull headgear. Informed consent was obtained from both the patients and their parents.

**Figure 1:**
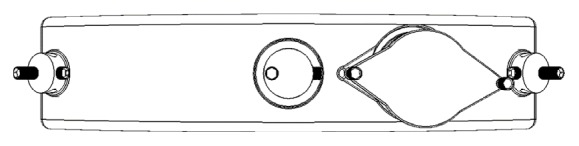
The new forehead design.

**Figure 2:**
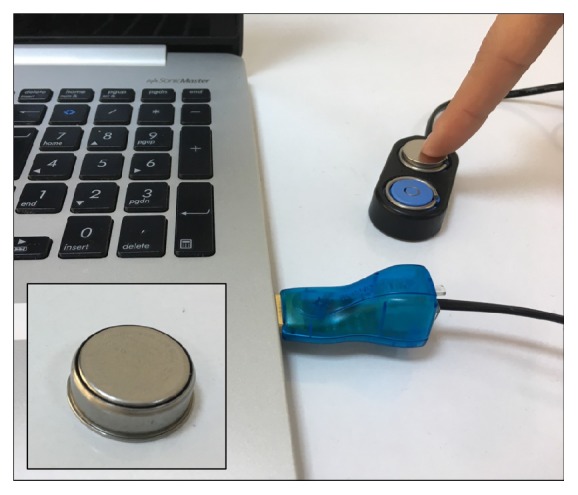
Sensor and programming process of sensor.

The newly designed headgear has a sensor slot in forehead part (Figure 1). The sensor of system (iButton) is a sophisticated digital thermometer (Figure 2).

The main function of the sensor is to measure the temperature and store the value of it in its memory [8].

**Figure 3:**
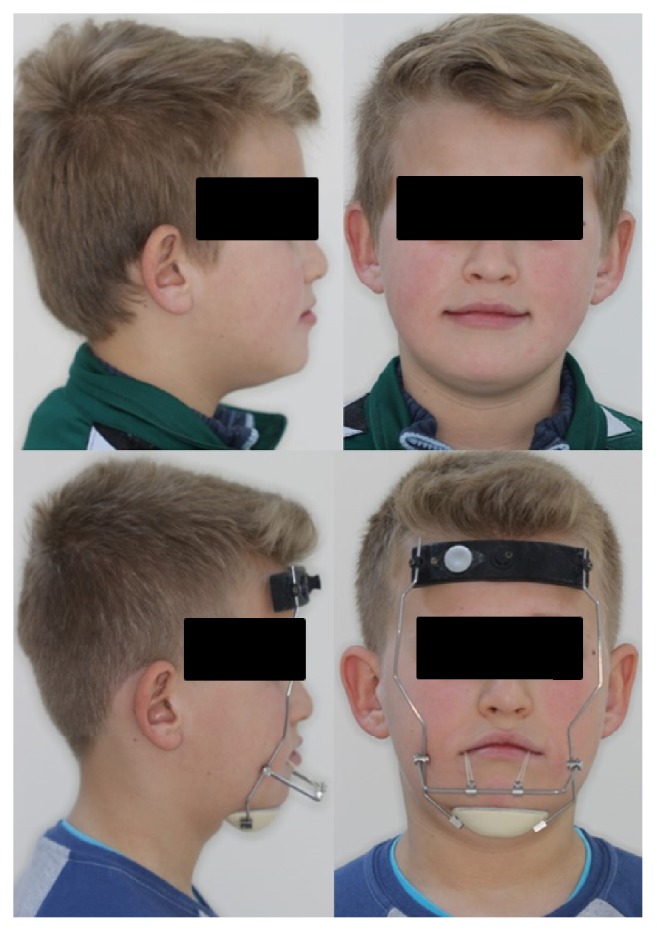
Application of force.

Study models with bands were taken from all patients for hooked hyrax device production. Four banded hooked hyrax device and extraoral elastics were used to provide adequate orthopedic force loading (Figure 3).

At the first treatment visit, hyraxes were placed in the patients' mouths and the reverse pull headgear was applied. Hyraxes were activated at the beginning of the reverse pull headgear therapy. Orthopedic force level (around 450 gr per side) was loaded with elastics and patients were asked to wear them for 13–16 hours per day, especially during the evening and night. Patients were given information about the increased release of growth hormone and other growth-promoting endocrine factors, which has been observed to be higher during the evening and night than during the day. However, patients could decide for themselves what time of the day they wore the headgear. Both patients and their parents were also informed about possible damage caused by facial trauma during daily activities such as sports. Patients and parents were asked to explain the therapy to their friends and teachers in order to prevent demotivational factors like teasing at school.

During study, 15 programmed sensors were placed on the newly designed forehead part of the reverse pull headgear. At the second treatment visit (4 weeks after first treatment visit), new reverse pull headgears were given to 15 patients (Figure 3). Patients were seen for 4-5 weeks. Data of new reverse pull headgear were collected and stored.

Clinical examinations of patients were made and all the orthodontic mechanics were checked carefully.

At the end of study, stored data of new reverse pull headgears were evaluated and statistical analyses were made. Different days of week factor, different hours of day factor, and sex factor were analyzed by using Chi-squared test and ANOVA with an associate post hoc analysis (Tukey) was also used in more detailed statistical evaluation.

## 3. Results

During the study, any damage was detected on new reverse pull headgears, sensor, and hooked hyrax devices. All the new reverse pull headgears worked well and provided data. Temperature values were approximately 16–28°C (mean 24 ± 3.1°C) while the reverse pull headgear was not worn and approximately 31–39°C (mean 36.3 ± 2.9°C) temperature values while being worn by patients. The new reverse pull headgears measured the temperature and stored the values at planned intervals.

The data analysis showed the following results.

**Figure 4:**
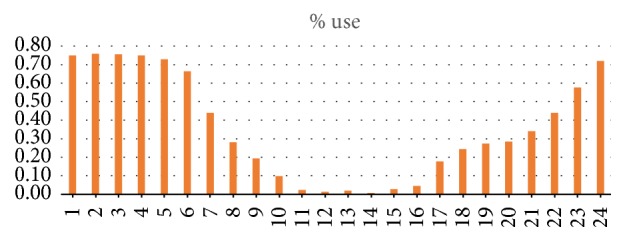
Graphics of all patients' usage percent during day (24 hours).

**Figure 5:**
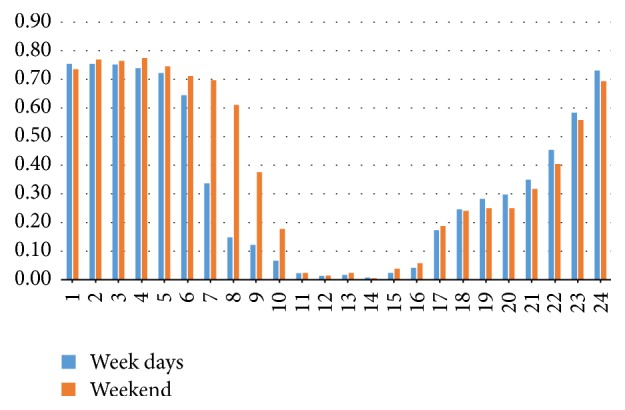
Graphics of comparison of usage percent between week days and weekends.

**Figure 6:**
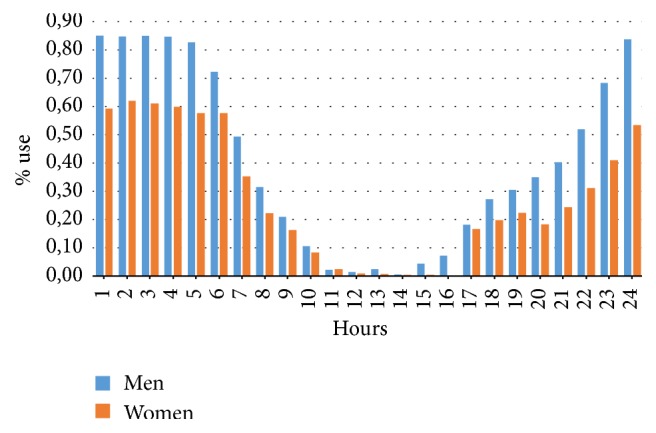
Reverse pull headgear usage percent according to hours of day and sex.

- The reverse pull headgear usage during night is significantly higher than daytime (*p* < 0.05) (Table 1) (Figure 4).
- The reverse pull headgear usage during evening is significantly higher than daytime (*p* < 0.05) (Table 1) (Figure 4).
- The reverse pull headgear usage during night is significantly higher than evening (*p* < 0.05) (Table 1) (Figure 4).
- Usage increases over weekend (*p* < 0.05) (Tables 2 and 3) (Figure 5).
- The usage habits of boys and girls are not alike at different hours of day (*p* < 0.05) (Tables 4 and 5) (Figure 6).

## 4. Discussion

The total wearing time and regularity of usage of reverse pull headgear therapy directly affects the success of these devices. An objective determination of the effect of reverse pull headgear can only be possible if the clinician obtains accurate information about headgear wear. Therefore, it is necessary to know the objective of reverse pull headgear usage when planning the treatment process. If the patient does not use the reverse pull headgear, clinicians can try repeatedly to motivate the patient. However, if the patient insists on resisting the suggestions, treatment can be delayed or other treatment alternatives can be considered. Thus, loss of money and time can be prevented.

Different techniques such as usage charts and talking with the patient and their parents, teachers, or school friends can provide information about compliance while undergoing reverse pull headgear therapy, but all provide subjective results. Obviously, there is a need for devices that provide objective measurements [9].

In modern orthodontics, reverse pull headgear was designed and made only for their mechanical requirements. They are produced to provide enough anchorage to generate the required protraction forces. A few studies used built-in electronic timing devices in removable appliances [10, 11]. In one study, DS1921G was used to measure patience compliance during cervical headgear therapy. Other studies investigated different devices such as a headgear timing device [12–14] and small quartz calendar [15]. These systems are not suitable to the aims of this study.

Reverse pull headgear therapy is indicated for patients with maxillary deficiency. The new reverse pull headgear was used for the same indication as the conventional one. Therefore, the evaluation of effectiveness of new reverse pull headgear can be achieved properly.

A hooked hyrax device and extraoral elastics were used to provide adequate orthopedic force loading. Orthopedic force level was loaded with elastics and 13–16-hour wearing time per day was required similar to that of routine reverse pull headgear therapy. The first month of treatment is the adaptation phase for patients and parents. Moreover, an evaluation of patients' behavior at this phase cannot provide sound results. So, at the second treatment visit, 15 patients began to use the new reverse pull headgear. The new reverse pull headgear could be used to track the patient' personal headgear wearing time. Sensors (iButton) have been used in different types of studies such as sleep evaluation or the body temperature measurement of mammals. The type of iButton used in the present study was coded H, meaning human. So the characteristics of this type of iButton are appropriate for human studies.

All patients and parents were aware that the new reverse pull headgear could identify and store the wearing times. Sound and objective communication between the family and clinician is one of the bullet benefits of new reverse pull headgear. Because this factor improves the truth of patient and parents reports about wearing reverse pull headgear, patients cannot claim that they have put on reverse pull headgears as recommended by clinician although they have not. This good, accurate, and true communication between family and clinician can possibly improve the quality of treatment.

New reverse pull headgear's data is provided to understand the patient's behavior. By the way, patients can be given a chance to explain their feelings about therapy and reasons of their behavior. All of the patients reported that they are not beautiful or handsome with reverse pull headgear. As a result, they did not want to wear appliances while they are among people like school times or social activities like birthday party. So results of the study such as popularity of use at weekend and night can be explained by poor aesthetics of reverse pull headgear. Patients prefer to use headgears at bed time or evening at home. All of these findings are expected results. Because many patients on daily practice of reverse pull headgear therapy also complain about aesthetic appearance of reverse pull headgear. This objective measurement of patient compliance is very important for clinicians on the way of the treatment, because clinician can delay or stop the treatment or apply other treatment options. Clinicians also can explain the benefits of treatment like good facial aesthetics and prevention from future surgery procedures to motivate the patients. All of these opportunities can be beneficial for family, clinician, and economy [16–18].

## 5. Conclusions

- Patients usually wear more often the reverse pull headgears at night and weekends, probably because of the poor aesthetic appearance of the appliance.
- Objective measurement of patient compliance can help clinicians with this challenging treatment.
- Although not a hypertechnological appliance, the new reverse pull headgear can be used successfully to monitor patient compliance.

## Figures and Tables

**Table 1:** Comparison of usage time according to different hours of day and sex (two-way ANOVA).

	Df	Sum Sq	Mean Sq	*p* value
Hour factor	23	1386.7	60.7	*∗∗∗*
Sex factor	1	68.9	68.91	*∗∗∗*

*∗∗∗* means *p* < 0.05.

**Table 2:** Comparison of days (*p* values).

Tukey multiple comparisons of means (95% family-wise confidence level)							
	Monday	Tuesday	Wednesday	Thursday	Friday	Saturday	Sunday
Monday		0.6115052	0.7447838	0.9955896	0.9999974	**0.0054993**	**0.0014632**
Tuesday			0.0236437	0.2217987	0.4879183	**0.0000024**	**0.0000004**
Wednesday				0.9769117	0.8384991	0.3473935	0.1787557
Thursday					0.9992788	**0.0437167**	**0.0148834**
Friday						**0.0099470**	**0.0028086**
Saturday							0.9998783
Sunday							

**Table 3:** Comparison of usage time according to different hours of day, different days of week, and sex (ANOVA).

	Df	Sum Sq	Mean Sq	*p* value
Hour factor	23	1386.7	60.29	*∗∗∗*
Week days/weekend factor	1	8.1	8.07	*∗∗∗*
Sex factor	1	69.0	68.97	*∗∗∗*

*∗∗∗* means *p* < 0.05.

**Table 4:** Comparison of male and female patients.

	Week days	Weekend
No		
Male	4771	1714
Female	3585	1318
Wear		
Male	3080	1358
Female	1296	603

Chi-square test of independence, number of cases in table: 17725, number of factors: 3, and test for independence of all factors: Chisq = 332.5, df = 4, *p* value = 1.066*e* − 70.

**Table 5:** Comparison of male and female patients.

		Monday	Tuesday	Wednesday	Thursday	Friday	Saturday	Sunday
Non	Male	987	1035	887	941	921	840	874
	Female	690	685	707	732	771	682	636
Wear	Male	602	532	652	637	657	696	662
	Female	270	275	253	266	232	279	324

Chi-square test of independence, number of cases in table: 17725, number of factors: 3, and test for independence of all factors: Chisq = 380.1, df = 19, *p* value = 5.953*e* − 69.
